# Study of physio-psychological effects on traffic wardens due to traffic noise pollution; exposure-effect relation

**DOI:** 10.1186/s40201-015-0187-x

**Published:** 2015-04-16

**Authors:** Shamas Tabraiz, Saeed Ahmad, Iffat Shehzadi, Muhammad Bilal Asif

**Affiliations:** Department of Environmental Engineering, University of Engineering & Technology, Taxila, Pakistan; Department of Psychology, University of Gujrat, Pakistan

**Keywords:** Noise pollution, Traffic wardens, Physio-psychological effects, Exposure-effect relation

## Abstract

**Background:**

Noise pollution has increased to alarming extent in most of the urban areas in Pakistan. It is assumed even more perilous than air and water pollution due to its direct acute and chronic physio-psychological effects. The objective of this study is to analyze and evaluate the psychological and physiological effects caused by traffic noise on traffic wardens and to find relation type between exposure time and effect.

**Methods:**

Three wardens check posts near roads were selected for survey in Taxila and Islamabad cities of Pakistan. Survey conducted included noise measurements at aforementioned check posts for one month and Performa based interviews of traffic wardens.

**Results and conclusions:**

Analysis of results showed that noise levels varied between 85-106 dB hence violating OSHA regulations. Major psychological effects found in wardens were aggravated depression 58%, stress 65%, public conflict 71%, irritation and annoyance 54%, behavioral affects 59% and speech interference 56%. Physiological effects found were hypertension 87%, muscle tension 64%, exhaustion 48%, low performance levels 55%, concentration loss 93%, hearing impairment 69%, headache 74% and cardiovascular issue 71%. Relation between exposure time and effects were evaluated by using simple regression test in excel. Percentage of psychological and physiological effects in wardens varied with the exposure time; aggravated depression (R^2^ = 0.946, P = 0.133), stress suffering (R^2^ = 0.014, P = 0.173), public conflict (R^2^ = 0.946, P = 0.133), irritation and annoyance (R^2^ = 0.371, P = 0.137), behavioral affects (R^2^ = 0.596, P = 0.0616) and speech interference (R^2^ = 0.355, P = 0.445), hypertension (R^2^ = 0.96, P = 0.00095) and cardiovascular issue (R^2^ = 0.775, P = 0.044).

## Introduction

Economical and social structure of society has been changed in Pakistan due to rapid population increase, urbanization and industrialization. Due to advancement in transportation facilities and technology the traffic loads are increasing at an alarming rate which initiates a major problem of noise pollution. Noise is an unwanted sound which has different frequencies and acoustic pressure without any regular pattern. The major causes of noise pollution are roads, railway and air traffic. Characteristics of noise (frequency and acoustic pressure) depend upon the characteristics of the traffic and road i.e. road gradient, road surface type, surrounding topography, grade of road, number of vehicles, type of vehicles, ages of vehicle passing, speed of vehicles, type of goods transported, packing of goods in vehicles, horns sound pressure, meteorological conditions, type of breaks and behavior of the drivers. Most effective way of controlling noise is to reduce it at source i.e. vehicle and road. Noise contribution from different parts of vehicles is different; Air intake system 9%, exhaust system 27%, tires 30% and engine 34%. At 70 km/h speed the tire noise dominate on other sources within the vehicle. Therefore, limiting the vehicle speed on busy roads substantially reduces noise the level [1]. Only 5% reduction in the gradient of road reduces 1.5 dB of Leq noise. Road surface macro-texture wavelength range should be 2-10-20 mm to reduce noise levels. Similarly, Green belts, plants and trees along the roads are effective in the reduction of noise levels. For 10 dB reduction, mounds and barriers along the roads are suggested to construct. Building treatment is also effective for the reduction of internal noise i.e. single glazed and double glazed windows reduce 10 and 25 dB noise level, respectively [2]. Management of noise is important to shun its perilous effects. High noise levels deteriorate the health of people and thus subjected to economy loss.

Access to pollution free environment is the central theme of human rights. Therefore, it should be everybody’s task to keep ambient environment natural and clean as much as possible [3]. Noise is discarded, unlikable and annoying sound of such duration, intensity or other quality that can cause any kind of physiological harm to human health or other living things [4]. Traffic noise is a source of annoyance [5-7]. It is detrimental to the health of every third person. Every one out of five persons in Europe is exposed to health deteriorating noise levels even at night (*WHO*). Noise has identified as one of the main environmental problem in Europe but lower priority is given to it as compared to water and air pollution [8]. A study was carried out on healthy individuals who migrated to the high and low noise levels residential areas of Bonn city. The blood pressure got to higher level in the individuals who moved to higher noise level areas as compared to those who moved in quitter areas [9]. Another study revealed that blood pressure quickly returned to normal when elevated noise came to lower level. Relationship between the noise levels and sleep quality is complex. It is recommended by World Health Organization that noise level in the bed room should be kept below the average level of 30 dB, or maximum of 45 dB for a single event. Higher levels of sound are related to reduced sleeping quality or awakening.

Cardiovascular effects due to traffic noise exposure are an important area of research in environmental epidemiology. Adverse health effects of noise like blood pressure and hypertension were studied. Noise level and blood pressure relationship found in both children and adults was inconsistent [10,11]. Rather, more consistent results have been obtained in case of hypertension, with aircraft noise as well as road traffic noise [12-22]. Relationship between exposure to traffic noise and hearing impairment, ischemic heart disease, hypertension, annoyance and sleep disturbance was established [23]. In the recent years the cause relationship between traffic noise and cardiovascular risk has been increased [24]. Impacts of noise pollution include but not limited to the impairment of mind-peace, internal peace, impairment of hearing and the physiological impacts [25]. Noise pollution could impair physiological and psychological aspects of human health [26]. The effects of long term exposure are not well understood. Long term effects are linked to hypertension and heart diseases [8]. Traffic wardens in the cities are mostly appointed on the traffic rush junctions of the roads to regulate the traffic. Therefore, they are exposed to elevated noise levels. Long term physio-psychological effects of traffic noise on traffic wardens and effects relation with exposure time has not been evaluated yet. Therefore, this study was undertaken with these objectives. Beside that Noise levels were also measured at three wardens check posts situated along the Grand Truck Road (Taxila, Islamabad).

## Materials and methods

The study was divided in two phases. In the first phase, noise levels were measured. In second phase, physio-psychological effects were evaluated.

### Exposure assessment

Three wardens check posts along the Grand Truck Road were selected for noise survey in Taxila and Islamabad. The check posts selected were Taxila underpass check post, Tarnol check post and Golra mor check post. These check posts were selected keeping in view the movement of crush and other goods carrying heavy vehicles. The measurements were taken at those points where the traffic wardens were found to be performing their duties. Usually they perform their duties within 1-5-10 m range from the road side. Noise monitoring was conducted for one month. The noise level meter during measurements was held at a height of 1.5 meter above ground level at an angle of 30° from monitoring persons’ body, to measure noise levels which can be perceived by the human ears. A one minute reading time was set at each location in order to obtain a stable maximum reading of noise at that particular location. In this way, 60 readings were noted at every location for an hour, each one after 1 minute then the Leq (8 hrs) for the day was calculated.

### Physio- psychological effects assessment

In the second phase, a wide-ranging standardized questionnaire was developed. It took 30-40 minute to interview a warden. Wardens appointed at the aforementioned three and nearby check posts were interviewed. The questionnaire covered housing (socio-economic conditions), exposure, heritage physio-psychological diseases, any accidental disability or psycho effect, age, working exposure, medication of any physio-psycho disease. The psychological effects evaluated were; aggravated depression, stress, public conflict, irritation and annoyance, behavioral effects and speech interference. The psychological effects were judged and evaluated on the basis of questions asked verbally: “Did you have aggravated depression? (Yes, no)”, “Did any one of your parents have aggravated depression? (Yes, no)”, “Did you have aggravated depression before joining the job as traffic warden? (Yes, no)”, “Did you ever take anti-depressive medicine? (Yes, No)”. Same types of questions were asked to assess other psychological effects. The physiological effects evaluated were; hypertension, muscle tension, exhaustion, exasperated fatigue, low performance levels, concentration loss, earache, hearing impairment, headache, cardiovascular issue, tinnitus and insomnia. The effects like muscle tension, exhaustion, exasperated fatigue, low performance levels, concentration loss, earache, headache, tinnitus and insomnia were evaluated with 7- grade verbal question; “Did you have hypertension? (Yes, no)”, “Did one of your parents have hypertension? (Yes, no)”, “Did you have hypertension before joining the job as traffic warden? (Yes, no)”, “Did you ever take anti-hypertensive medicine? (Yes, no)”, “Do you feel muscle tension during or after job hours? (Often, sometime, seldom or never)”, “Is there any reason of muscle tension other than noise at your job place? (Yes, no)”, if “Yes” then what? Same types of questions were asked to evaluate the effects like exhaustion, exasperated fatigue, low performance levels and concentration loss. For earache and headache; “Do you feel earache and headache due to high noise levels? (Often, sometime, seldom or never)”, “Do you ever got injury in head or ear? (Yes, no)”. For hearing impairment; “Do you have hearing impairment? (Yes, no)”, “Was there an accident which impaired your hearing? (Yes, no)”. For insomnia and tinnitus; “Do you have any sleeping problem (early wakeup, falling asleep, awakening)?: (often, sometime, seldom or never)” [27], “Do you have tinnitus problem (ear ringing)? (often, sometime, seldom or never)”. For the cardiovascular problem; “Do you have any cardiovascular issue (valve problem, ischemic stroke, heart failure)? (Yes, no)”. If yes, do you have heart problem before the joining of job? (Yes, no), “Are you taking any medication? (Yes, no)”. All the wardens were divided into different groups based on the job experience (exposure time); group A (1-5 years), Group B (5-10 years), Group C (10-15 years) and Group D (15-20 years). After the assessment of exposure and physio-psychological effects, the simple linear regression was applied by using Microsoft excel to estimate the association of exposure time with physio-psychological effects. Two hundred and fifty wardens were interviewed to evaluate the effects.

## Results and discussions

### Noise levels

**Figure 1:**
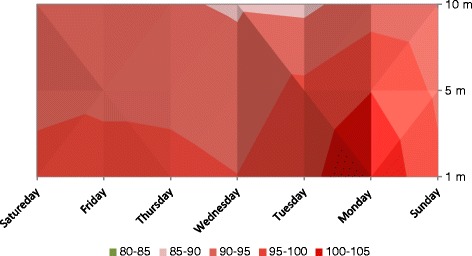
Noise levels (Leq 8hrs) at Taxila Underpass check post.

**Figure 2:**
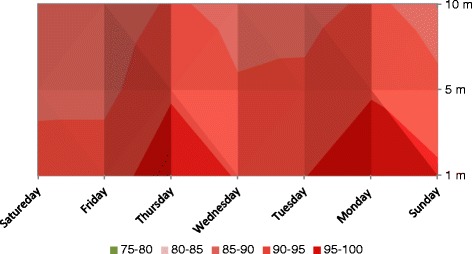
Noise levels (Leq 8hrs) Tarnol check post.

**Figure 3:**
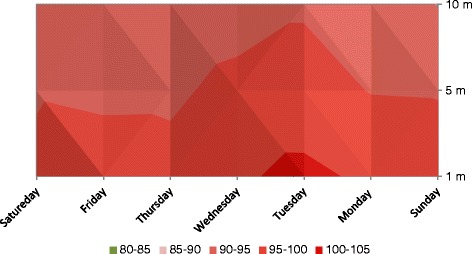
Noise levels (Leq 8hrs) Golra mor check post.

Noise level measurements were taken at selected locations near the check posts. Noise levels (Leq for 8 hours) of three check posts at 1m, 5 m and 10 m distance from road are given below in Figures 1, 2 and 3. Noise levels (Leq) varied from 86-106 dB. Such high levels were due to the heavy traffic and high pressure horns. Impulsive noise levels measured were up to 120 dB. Even at the distance of 10 m noise levels (Leq) did not dropped below 85 dB. At the distance of 10 m, noise levels range was 85-95 dB, at all the selected posts. While at the distance of 5 m from road, noise levels (Leq) measured were in the range 88-102 dB, at all the selected point. At 1m away from road, noise levels (Leq) range was 91-106 dB. Hearing impairment could be possible of such high noise pressure level (120 dB), even for short period (seconds) exposure. From the measurements, it was assessed that not only the wardens performing duty but people living near roads are under severe threat of possible physiological as well psychological effects.

### Psychological effects

**Figure 4:**
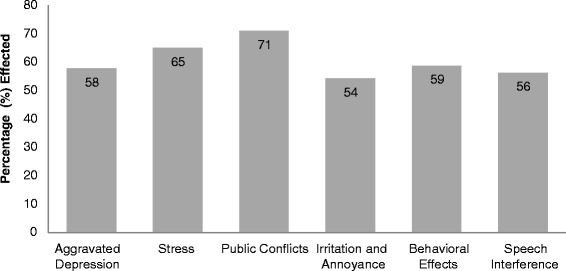
Psychological effects of noise on traffic wardens.

**Figure 5:**
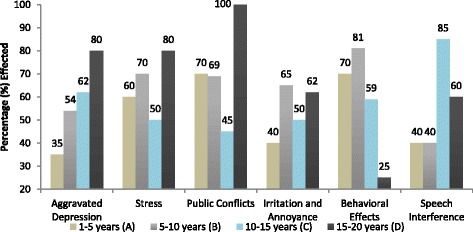
Exposure time wise psychological effects of noise on traffic wardens.

The common psychological effects i.e. hypertension, aggravated depression, stress, public conflict, irritation and annoyance, behavioral effects and speech interference were taken into account for this study. All the results of psychological effects are explained below and shown in Figures 4 and 5.

#### Aggravated depression

In aggravated depression one feels sadness, hopelessness and loss of interest in life. The survey study statistics showed that 58% of the wardens were suffering from aggravated depression. Exposure time wise, there was variance in effected warden’s percentage. Aggravated depression found; 35%, 54%, 62% and 80% in group A, B, C and D, respectively. A linear relation was found between exposure time and the percentage of aggravated depression suffering wardens (R^2^ = 0.978, P = 0.033).

#### Stress

In case of stress, 65% of the wardens of all the groups were suffering from stress. Exposure wise trend was random. Group A, B, C & D reported 60%, 70%, 50% and 80% stress, respectively. A non-linear relation was found between the exposure time and wardens percentage of stress suffering (R^2^ = 0.16, P = 0.093).

#### Public conflict

Public conflict evaluated was 71%. It showed random trend as the exposure time increased. 70%, 69%, 45% and 100% public conflict problem found for group A, B, C and D group, respectively. A non-linear relation was found between exposure time and the percentage of wardens reported public conflict often (R^2^ = 0.143, P = 0.223).

#### Irritation and annoyance

Irritation and annoyance was found in 54% wardens. Exposure time wise percentage suffering from irritation and annoyance was; 40%, 65%, 50% and 62% in group A, B, C and D, respectively. A non linear relationship was found between exposure time and percentage of wardens suffered irritation and annoyance (R^2^ = 0.327, P = 0.099).

#### Behavioral effects

Behavior changes were reported by 59% wardens. Exposure time wise behavioral effects reported were 70%, 81%, 59% and 25% in group A, B, C and D, respectively. Percentage effected showed a linear relation with exposure time (R^2^ = 0.7, P = 0.0388). Results showed that percentage increased initially from 1-10 years of exposure then decreased afterward.

#### Speech interference

56% of the surveyed wardens reported that noise create difficulty in conversation. Exposure time wise percentage of wardens’ reported speech interference was; 40%, 40%, 85% and 60% in group A, B, C and D, respectively. A non-linear relation was established between exposure time and the percentage of wardens reported speech interference (R^2^ = 0.4, P = 0.349).

### Physiological effects

**Figure 6:**
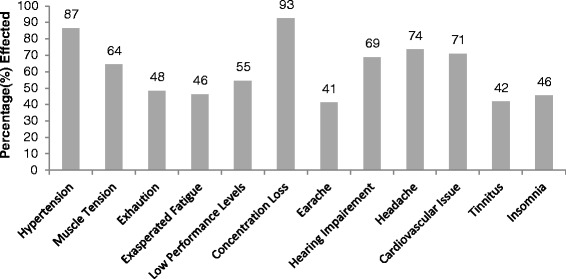
Physiological effects of noise on traffic wardens.

**Figure 7:**
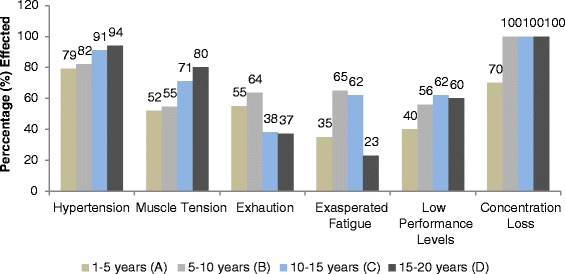
Exposure time wise Physiological effects of noise on traffic wardens.

**Figure 8:**
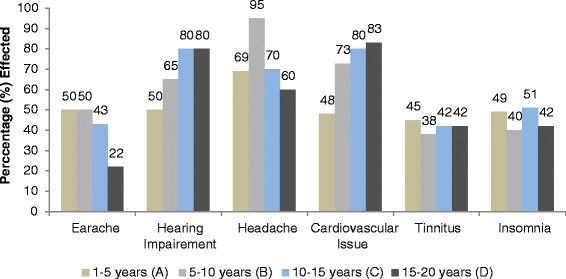
Exposure time wise Physiological effects of noise on traffic wardens.

In Figure 6, physiological effects that were investigated in this study for traffic wardens are given. Hypertension, muscle tension, exhaustion, exasperated fatigue, low performance levels, concentration loss, earache, hearing impairment, headache, cardiovascular issue, tinnitus and insomnia were probed. All the results of physiological effects are briefly discussed below and shown in Figure 6. Exposure time wise effects are shown in Figures 7 and 8.

#### Hypertension

In the survey 87% of the wardens were found to be suffering from hypertension (high blood pressures). The effected personnel percentile variation with the exposure time was as: in group A; 79%, group B; 82%, group C; 91% and group D; 94%. A linear trend was found between the exposure time and hypertension in the wardens (R^2^ = 0.976, P = 0.001).

#### Muscle tension

Muscle tension reported was 64%. Exposure time wise muscle tension reported by group A, B, C and D were 52%, 55%, 71% and 80%, respectively. It increased as the exposure time increased. A linear relation was found between wardens’ percentage affected and exposure time (R^2^ = 0.945, P = 0.0136).

#### Exhaustion

Survey results showed that 48% wardens were suffering from exhaustion. Exposure time wise exhaustion in group A, B, C and D was 55%, 64%, 38% and 37%, respectively. Percentage effected showed a little linear relation with exposure time (R^2^ = 0.609, P = 0.0312). Results showed that percentage reported exhaustion increased initially from 1-10 years of exposure then decreased afterward.

#### Exasperated fatigue

“Noise does exasperated fatigue” reported by 46% wardens. Exasperated fatigue found as 35, 65, 62 and 23% in group A, B, C and D, respectively. Percentage reported exasperated fatigue showed a non-linear relation with exposure time (R^2^ = 0.06, P = 0.201). Results showed that percentage increased initially from 1-15 years of exposure then decreased afterward up to 20 years exposure.

#### Low performance levels

“Elevated noise levels reduce the performance levels” reported by 55% traffic wardens. Group A, B, C and D were suffering low performance level; 40%, 56%, 62% and 60%, correspondingly. With the increase in exposure time the percentage effected increased. It showed linear relation (R^2^ = 0.728, P = 0.0396) between exposure time and percentage of effected wardens.

#### Concentration loss

Elevated noise caused concentration loss, 93% wardens reported. Exposure time wise concentration loss reported was 70%, 100%, 100% and 100% in group A, B, C and D, respectively. After 5 years of exposure 100% personnel reported concentration loss due to noise. It showed approximately constant relation after 5 years (R^2^ = 0.6, P = 0.0389) between exposure time and percentage of effected wardens.

#### Earache

Only 41% wardens were suffering from earache due to the elevated noise levels. Exposure wise wardens suffering earache; 50%, 50%, 43% and 22% in group A, B, C and D, respectively. A linear relation (R^2^ = 0.786, P = 0.020) was found between warden’s earache reported percentage and exposure time. Earache decreased with increase in exposure time.

#### Hearing impairment

Hearing capability has been impaired of 69% wardens. Hearing impairment increased as the exposure time increased. The effected personnel percentile variation with the exposure time was as: in group A; 50%, group B; 65%, group C; 80% and group D; 80%. A linear trend was found between the exposure time and hearing impairment in the wardens (R^2^ = 0.896, P = 0.0269).

#### Headache

Headache was reported by 74% traffic wardens. Headache increased and then decreased after ten years time period. Exposure time wise headache; in group A, B, C and D E was 69%, 95%, 70% and 60% respectively. Initial it increased to 10 years after that it decreased. A non linear relation (R^2^ = 0.199, P = 0.0502) between exposure time and headache in warden was found.

#### Cardiovascular issue

On the whole, 71% wardens reported cardiovascular issues. Exposure time wise Cardiovascular issues; in group A, B, C and D was 48%, 73%, 80% and 83%, respectively. A linear relation (R^2^ = 0.827, P = 0.049) between exposure time and cardiovascular issues in wardens was found. Heart problem increased as the exposure to noise increased.

#### Tinnitus

Ringing ear reported by 42% wardens only. This percentage was approximately constant (R^2^ = 0.05, P = 0.0039) in each group A, B, C and D.

#### Insomnia

About 46% wardens feel difficulty in sleeping (early wakeup, falling asleep, awakening). Exposure time wise insomnia found in group A, B, C and D was 49%, 40%, 51% and 42% respectively. A non- linear relation (R^2^ = 0.00588, P = 0.025) between exposure and insomnia was established. Summary of the regression test for exposure effect relation is given below in Table 1.

**Table 1:** **Summary of simple regression statistics (exposure-effect relation)**

**Physio-psychological effects**	**(R** ^**2**^ )*	P-value*	**Exposure-effect relation**
**Aggravated Depression**	0.978	0.033	Linear
**Stress**	0.16	0.093	Non-linear
**Public Conflicts**	0.143	0.233	Non-linear
**Irritation and Annoyance**	0.327	0.099	Non-linear
**Behavioral Effects**	0.7	0.0388	Non-linear
**Speech Interference**	0.4	0.349	Non-linear
**Hypertension**	0.976	0.001	Linear
**Muscle Tension**	0.945	0.0136	Linear
**Exhaustion**	0.609	0.0312	Linear
**Exasperated Fatigue**	0.06	0.21	Non-linear
**Low Performance Levels**	0.728	0.0396	Linear
**Concentration Loss**	0.6	0.089	Linear
**Earache**	0.786	0.020	Linear
**Hearing Impairment**	0.896	0.0269	Linear
**Headache**	0.199	0.052	Non-linear
**Cardiovascular Issue**	0.827	0.049	Linear
**Tinnitus**	0.05	0.0039	Constant
**Insomnia**	0.00588	0.025	Non-linear

*P-value is the probability of observing a test statistic more extreme than what was observed (if p < 0.05 then null hypothesis rejected).

*R^2^ is a coefficient of determination (measures the how accurate linear model is at predicting i.e. near to unity mean more linear relation).

## Conclusions

Noise pollution at selected locations has increased up to dangerous levels. Noise pollution is one of major cause of physiological and psychological deterioration but never gets due attention. Some of the physio-psychological effects showed high percentage of effected traffic wardens initially but decreased as the exposure time increased i.e. behavioral effects, speech interference, exhaustion, exasperated fatigue, ear each and head each. Most of the physio-psychological effects increased as the exposure time increased i.e. hypertension, aggravated depression, muscle tension, low performance levels, concentration loss, hearing impairment and cardiovascular issues. Few effects remained constant as the exposure time increased tinnitus and insomnia, while some effects showed random trends with increase in exposure time i.e. irritation and annoyance, public conflicts and stress. Further investigation of the effects is recommended which showed the random relation with exposure time to reveal reasons.

It can be concluded from the results that physiological and psychological deterioration lead to disturbed social life of traffic wardens. Therefore, efforts should be done to provide a framework in order to reduce duty hours for traffic wardens based on their physiological deterioration due to prolong exposure. Traffic wardens should be equipped with personal protective equipments such as ear plugs to protect their hearing impairment. Most importantly noise reduction techniques should be applied while the road planning, design and construction is in process. Legislation for the standard of vehicles should be in Pakistan. Enforcement of present rules regarding noise could help in noise pollution reduction.
